# Highly Efficient Quasi-Solid-State Asymmetric Supercapacitors Based on MoS_2_/MWCNT and PANI/MWCNT Composite Electrodes

**DOI:** 10.1186/s11671-019-2902-5

**Published:** 2019-02-26

**Authors:** Bing Cheng, Renzhi Cheng, Furui Tan, Xiansheng Liu, Jinghao Huo, Gentian Yue

**Affiliations:** 1 Henan Key Laboratory of Photovoltaic Materials and Laboratory of Low-Dimensional Materials Science, Henan University, Kaifeng, 475004 China; 2 School of Physics and Electronics, Henan University, Kaifeng, 475004 China; 3 School of Materials Science and Engineering, Shaanxi University of Science and Technology, Xi’an, 710021 China

**Keywords:** Molybdenum disulfide, Multi-walled carbon nanotube, Polyaniline, Quasi-solid-state, Supercapacitor

## Abstract

Molybdenum disulfide (MoS_2_) and polyaniline (PANI) electrodes were decorated with multi-walled carbon nanotubes (MWCNTs) on the basis of a facial hydrothermal and in situ polymerization methods and served in the asymmetric supercapacitor (ASC). The MoS_2_ and MWCNTs with a mole ratio of 1:1 in MoS_2_|MWCNTs electrode exhibited better electrochemical properties through extensive electrochemical studies, in terms of the highest specific capacitance of 255.8 F/g at 1 A/g, low internal resistance, and notable electrochemical stability with retention of the initial specific capacitance at 91.6% after 1000 cycles. The as-prepared PANI|MWCNTs electrode also exhibited good specific capacitance of 267.5 F/g at 1 A/g and remained 97.9% capacitance retention after 1000 cycles. Then, the ASC with MoS_2_|MWCNTs and PANI|MWCNTs composite electrodes were assembled with polyvinyl alcohol (PVA)-Na_2_SO_4_ gel electrolyte, which displayed good electrochemical performance with the specific capacitance of 138.1 F/g at 1 A/g, and remained the energy density of 15.09 Wh/kg at a high power density of 2217.95 W/kg. This result shows that this ASC device possesses excellent electrochemical properties of high energy density and power output and thus showing a potential application prospect.

## Background

With the energy crisis and environmental pollution increasing seriously, the efficient and stable energy storage equipment has attracted extensive research due to the intermittent energy sources (such as solar energy, wind power, and frictional power) cannot continuously and stably output electrical energy for daily application [1, 2]. Supercapacitors (SCs) with fast charging/discharging and long cycling life are considered as an ideal choice for energy storage devices, which overall performance is mainly determined by the most important components, i.e., electrode materials [3]. Typically, a significant amount of electrode materials, like graphene, carbon nanotubes (CNTs), conducting polymers, and oxide metals, have become a research focus [4–7]. In particular, pseudocapacitive materials with remarkably high capacitance have attracted a large number of concerns [8].

Among these reports, carbon materials are identified as one of the most popular because of their excellent conductivity, large surface area, environmental friendly protecting, and low cost. However, the carbon materials store charges by electric double-layer capacitor mechanism possess high electrical conductivity but low capacitance. Thus, to improve the electrochemical performance of carbon-based materials, researchers have devoted great efforts to grow nano-structured active materials on them [9–15]. Molybdenum disulfide (MoS_2_), as a typical family member of transition-metal dichalcogenides (TMDs), has attracted lots of attention for its special structural and chemical character, which is extensively applied in many fields including lithium-ion batteries, catalysis, and dye-sensitized solar cells. MoS_2_ with nanoscale recently has been chosen to use in capacitors owing to its higher intrinsic fast ionic conductivity than oxides and higher theoretical capacity than graphite besides high surface area. Wang et al. [16] prepared MoS_2_ with hierarchical hollow nanospheres structure as negative electrode material providing a maximum specific capacitance of 142 F/g at 0.59 A/g. Yang et al. [17] designed MoS_2_/graphene nanosheet composites and obtained a specific capacitance of 320 F/g at 2 A/g. Hu et al. [18] prepared porous C|MoS_2_ electrode which obtained the specific capacity of 210 F/g at 1 A/g and good stability with more than 1000 cycles at 4 A/g. However, the poor specific capacitance of MoS_2_ attributed to the stacking and collapse in the charge-discharge process of its two-dimensional layered structures, which limits the application in SCs. Therefore, how to construct hierarchical three-dimensional (3D) architectures is an effective way to solve the problem of aggregation and prepare high-performance supercapacitors based on MoS_2_ composites electrode.

In addition, with reported good conductivity, ease of synthesis, low-cost monomer, tunable properties, and remarkable specific capacitance (500–2200 F/g), polyaniline (PANI) has been extensively studied as a pseudo-supercapacitor electrode material [19–21]. Recently, many researchers have combined PANI with carbon materials and transition metal compound to improve the conductivity of the PANI-based electrodes for enhancing the cycling stability and rate capability of the PANI-based pseudo-super-capacitors [22–27]. For example, Li and his workers [28] prepared 3D CNTs|PANI fibers and obtained a specific capacitance as high as 242.9 F/cm in 1 M H_2_SO_4_ electrolyte. Das et al. [29] reported an asymmetric supercapacitor (ASC) assembled with a Prussian blue/MnO_2_-positive electrode and a PANI/graphene nanoplatelet composite as the negative electrode in KNO_3_ electrolyte and exhibited manifested favorable specific capacitance of 98 F/g at 1 A/g. Ghosh et al. [30] have fabricated high-energy density all-solid-state flexible ASC by using a facile novel 3D hollow urchin-shaped coaxial MnO_2_@PANI composite as positive electrode and 3D graphene foam as negative electrode materials with polyvinyl alcohol (PVA)/KOH gel electrolyte. Conductive PANI not only works as the bridge between MnO_2_ and graphene to enhance the electrical conductivity, but also improves the specific capacitance of the electrode. Moreover, recent studies also exhibited that gel electrolyte has revealed the most potential application prospects for supercapacitors [31, 32].

In view of these, we prepared MoS_2_ bridged by multi-walled carbon nanotubes (MWCNTs) through a facile hydrothermal method and prepared the PANI decorated with MWCNTs composite by using in situ chemical polymerization process. After extensive electrochemical testing, the specific capacity of MoS_2_|MWCNTs and PANI|MWCNTs electrodes obtained 255.8 F/g and 267.5 F/g at 1 A/g, respectively. The retention rate of the MoS_2_|MWCNTs electrode retained 91.6% after 1000 cycles at the scan rate of 30 mV/s. Also, the PANI|MWCNTs electrode also exhibited 97.9% retention at the scan rate of 60 mV/s after 1000 cycles. A quasi-solid-state ASC based on MoS_2_|MWCNTs and PANI|MWCNTs electrodes with PVA-sodium sulfate (Na_2_SO_4_) gel electrolyte showed energy density of 38.9 Wh/kg at the power density of 382.61 W/kg. Two such super quasi-solid-state ASC in series can easily light a red light-emitting diode, indicating potential application prospects.

## Methods

### Synthesis of MoS_2_|MWCNTs

The preparation of MoS_2_ is done by using a simple hydrothermal method [33]. Firstly, 0.726 g of sodium molybdate and 0.684 g of thiourea were mixed together in 35 ml of deionized water by stirring and sonicating for 30 min successively. Then, a certain amount of MWCNTs with different contents was added into the above mixture and sonicated for another 30 min. Subsequently, the mixed solution was adjusted the PH to less than 1 with 12 M hydrochloric acid. After that, the solution was transferred into a 50-ml Teflon liner and heated at 200 °C for 24 h without intentional control of ramping or cooling rate. When the temperature cooled down to room temperature, the precipitation was collected by filter and washed with ethanol and distilled water for five times, and then dried in a vacuum oven at 60 °C for 24 h. Furthermore, the mole ratios of MoS_2_ and MWCNTs with 2:1, 1:1, and 1:2 were also researched and labeled as MS2MWCNT1, MS1MWCNT1, and MS1MWCNT2 respectively. For comparison, pure MoS_2_ and MWCNTs were labeled as MS1MWCNT0 and MS0MWCNT1, respectively.

### Synthesis of PANI|MWCNTs

The preparation of PANI|MWCNTs was based on the previous reported [34, 35]. Firstly, 18 mg of MWCNTs were dispersed in 10 ml of deionized water and sonicated for 0.5 h and labeled as solution A. Then, 0.3 ml of aniline monomer was dissolved in 10 ml of 1 M hydrochloric acid and labeled as solution B. Subsequently, 0.21 g of potassium persulfate was dissolved in 10 ml of 1 M hydrochloric acid and labeled as solution C. Afterwards, solution B was added to solution A under magnetic stirring followed by sequential addition of solution C drop by drop, until the mixed solution turned dark green. At room temperature, this reaction continued for more than 5 h. After that, the product was collected by centrifugation and washed repeatedly with deionized water and absolute ethanol. The as-prepared sample was labeled as PANI|MWCNTs.

### Preparation of Quasi-Solid-State ASC

The quasi-solid-state ASC assembled as a sandwich structure by clipping a MoS_2_|MWCNTs cathode and a PANI|MWCNTs anode with PVA-Na_2_SO_4_ quasi-solid-state electrolyte, and the ASCs were labeled as MoS_2_|MWCNTs//PANI|MWCNTs (MM//PM). Firstly, the electrode material slurry as the mass ratio of active material (MoS_2_|MWCNTs and PANI|MWCNTs composites), nano graphite powder, and PVDF is 75:15:10 in NMP solvent, which was loaded on foam nickel to be pressed by using a doctor blade method. Prior to testing, the electrode material was dried in a vacuum oven at 60 °C for 12 h, and then immersed in the 0.5 M Na_2_SO_4_ electrolyte for 12 h. To prepare the PVA-Na_2_SO_4_ gel, 2 g of PVA was dissolved in deionized water at 90 °C and then 0.5 M Na_2_SO_4_ was added under vigorous stirring to obtain a clear solution. The gel was allowed to cool to room temperature after which it was used to fabricate the ASC.

### Characterization and Electrochemical Measurement

The surface morphologies of the samples were observed by using JSM-7001F field emission scanning electron microscope (SEM). The crystalline structures of the composites were investigated by glancing incident X-ray diffractometer (X‘Pert Pro, PANalytical B.V., Netherlands). Raman scattering was collected on a Renishaw RW1000 confocal microscope with 514 nm line of Ar + iron laser as the exciting light.

Cyclic voltammetry (CV) measurements were conducted in a three-electrode one-compartment cell, in which an as-prepared sample electrode was taken as the working electrode, a Pt sheet of 1.5 cm^2^ as CE, and an Ag/AgCl electrode as a reference electrode in 6 M aqueous KOH solution. The electrochemical impendence spectroscopy (EIS) tests were carried out simulating open-circuit conditions at ambient atmosphere by using an electrochemical measurement system (CHI660E, Shanghai Chenhua Device Company, China) at a constant temperature of 20 °C with AC signal amplitude of 20 mV in the frequency range from 0.1 to 10^5^ Hz at 0 V DC bias in the dark. The galvanostatic current charge-discharge (GCD) curves were conducted using a computer-controlled electrochemical analyzer (CHI 660E, CH Instrument). The specific capacitance (*Cs*) of the supercapacitor was calculated according to the following equations [36–39]:

1 $$ {C}_s\kern0.5em =\kern0.5em \frac{4\times \Delta t}{\Delta V\times {m}_{\mathrm{ac}}} $$

where *I* represents the current density (A), *Δt* represents the discharge time (s), *ΔV* represents the working potential window (V), *m*_ac_ represents the quality of active materials (g).

## Results and Discussion

### Cathode Material: MoS_2_|MWCNTs

**Fig. 1:**
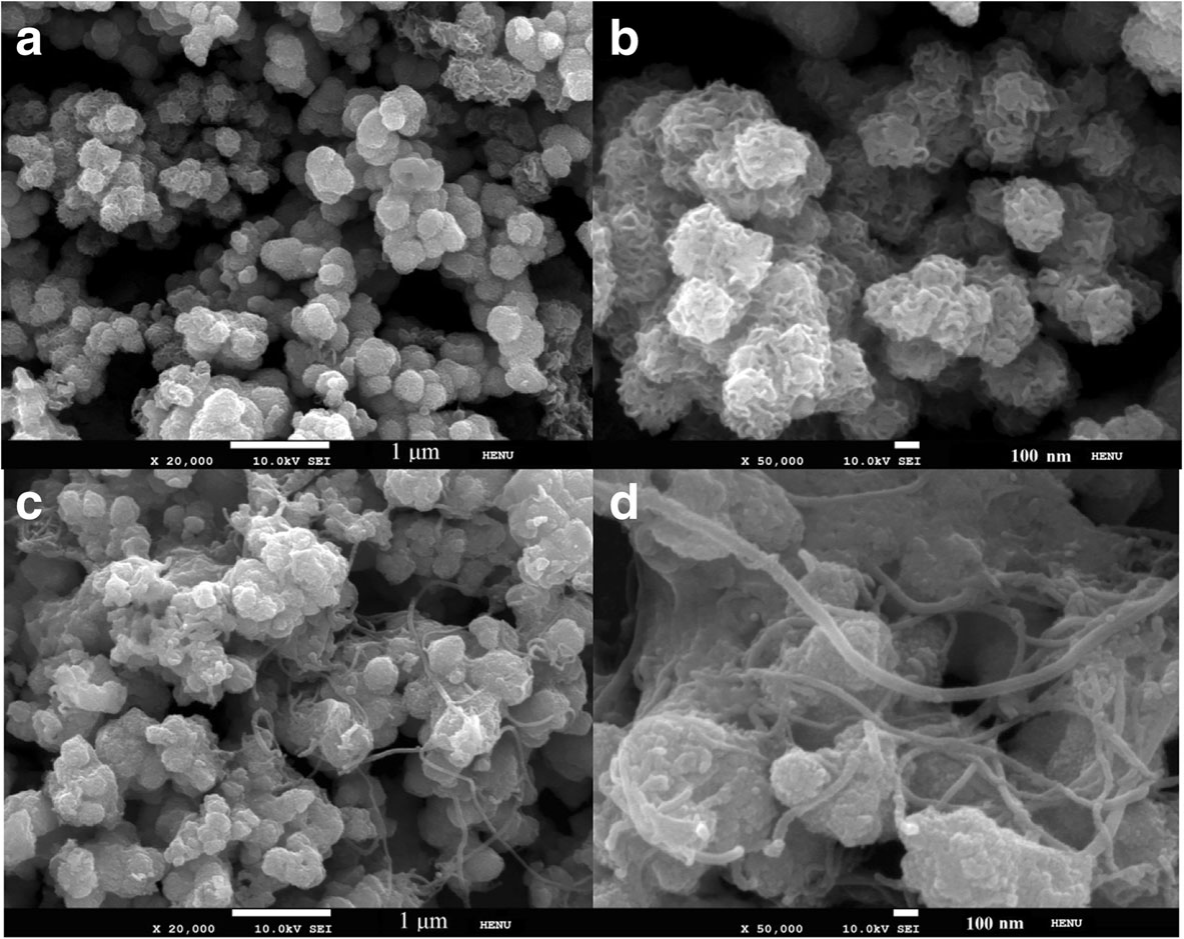
The SEM images of the MoS_2_ nanospheres (**a**, **b**) and the composite of MS1MWCNT1 (**c**, **d**)

Figure 1 shows the SEM images of the MoS_2_ and MS1MWCNT1 composite. From Fig. 1a and Fig. 1b, it can be seen that the synthesis of MoS_2_ nanospheres with honeycomb structure has a uniform distribution and similar particle size. From Fig. 1b, it exhibits that the surface of MoS_2_ nanospheres has many wrinkles, which are due to the stacking of the MoS_2_ nanosheets resulting in agglomerated spheres. Such a structure not only helps to provide large specific surface area, but also contributes to the diffusion and transfer of ions. Figure 1c and Fig. 1d show the images of MS1MWCNT1 composite, in which the MoS_2_ nanoclusters are bridged together by MWCNTs and form the MS1MWCNT1 composite. The MWCNTs have good conductivity and large specific surface area, which can compensate for the poor conductivity of MoS_2_ and provide more active sites by the edge of MWCNTs.

**Fig. 2:**
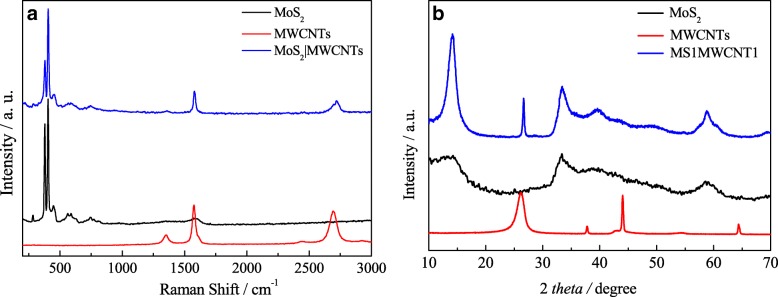
Raman spectrum (**a**) and XRD patterns (**b**) of MWCNTs, MoS_2_, and MS1MWCNT1

The structural characteristics of the as-prepared MoS_2_, MWCNTs, and MS1MWCNT1 were exhibited by Raman spectrum and XRD patterns. From Fig. 2a, there are two strong and sharp peaks at 375 and 408 cm^−1^ for MoS_2_. The former characteristic peak is called the *E*_2g_ mode due to the in-plane vibration of the two *S* atoms relative to the Mo atom in the opposite direction. The latter characteristic peak is called the *A*_1g_ mode for the non-plane *S* atom vibration in the opposite direction [40, 41]. For the Raman characteristic peaks of MWCNTs, the *D* and *G* peaks are around 1350 and 1580 cm^−1^, respectively [42, 43]. From these peaks ratios, we can see that the MWCNTs have good crystal purity and defect density. For the MS1MWCNT1 composite, the characteristic peaks of MoS_2_ and MWCNTs all exist and there are no new peaks emerge except a little red shift. The phenomenon of a little red shift for the MS1MWCNT1 composite can be responsible for the change of particle size and pore diameter. On the whole, this result indicates that the MS1MWCNT1 is successfully prepared without new compound formation.

Figure 2b shows XRD patterns of MoS_2_, MWCNTs, and MS1MWCNT1 samples. The diffraction peaks at 14.46°, 33.28°, and 58.66° are the contributions of MoS_2_ [44].9 Among them, the absence of strong diffraction peak of MoS_2_ reveals that stacking of the single layers very possibly takes place, which is named graphene-like structure. The diffraction peaks at 26.09° and 43.44° correspond to the signals of MWCNTs [45]. As for the XRD patterns of the MS1MWCNT1 composite, it exhibits all the characteristics of diffraction peaks for the MWCNT and MoS_2_. Especially, the strong peak at 14.46° of the MoS_2_ manifests a well-stacked layered structure, which demonstrates that the crystallinity of MoS_2_ is greatly improved after recombination. The Raman and XRD results indicate that the MoS_2_ and MWCNTs composite are synthesized successfully.

**Fig. 3:**
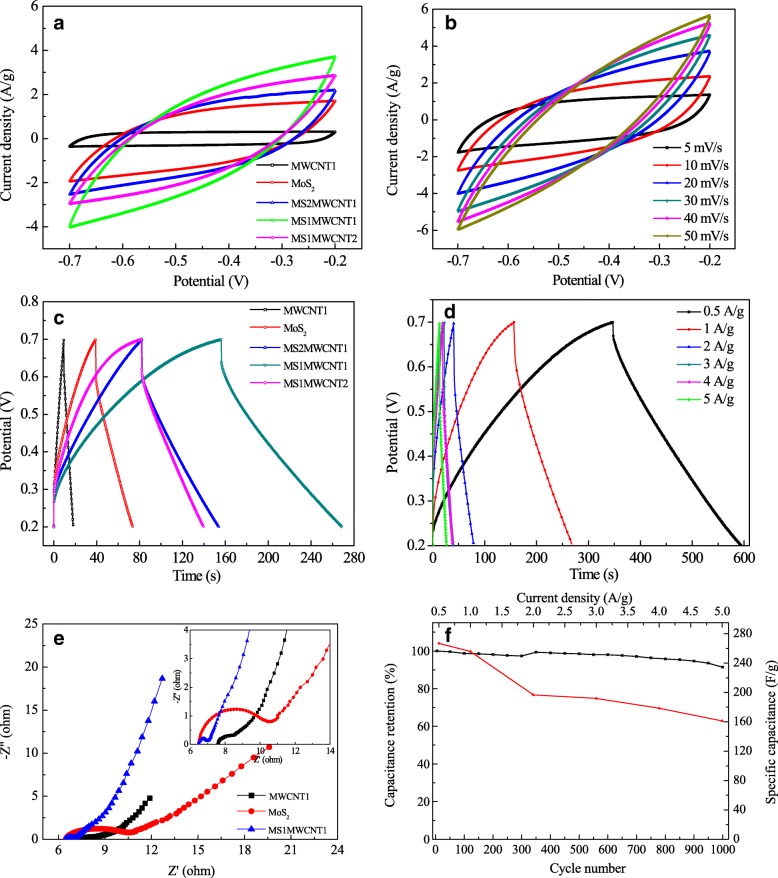
CV curves for the MoS_2_|MWCNTs electrodes with different mole ratios of MoS_2_ and MWCNTs at 20 mV/s (**a**), CV curves of MS1MWCNT1 at different scan speeds (**b**), GCD curves for the different electrodes at 1 A/g (**c**), GCD curves of MS1MWCNT1 with different current densities (**d**), EIS curves for the various electrodes (**e**), the stability after 1000 cycles CVs (black line), and specific capacitance at different current densities (red line) of the MS1MWCNT1 electrode (**f**)

The electrochemical characteristics of the as-prepared MoS_2_|MWCNTs electrodes are measured by CV, GCD, and EIS tests in Fig. 3. Figure 3a shows the CV curves for the MoS_2_|MWCNTs electrodes with different mole ratios of MoS_2_ and MWCNTs at 20 mV/s. The integrated CV area of the MoS_2_|MWCNTs electrodes shows larger than the pristine MWCNT and MoS_2_ electrodes, indicating the capacitance improvement for the synergistic effect from the excellent conductivity of MWCNTs and the high electrochemical performance of MoS_2_. Similarly, the MS1MWCNT1 electrode presents the largest CV area than that of the MS1MWCNT2 and MS2MWCNT1 electrodes, indicating excellent pseudocapacitive behavior for the MS1MWCNT1 electrode. In addition, the CV curves of the MS1MWCNT1 electrode with different scan rates ranging from 5 to 50 mV/s are tested as shown in Fig. 3b. As we can see from the curves, the current density and redox peaks increase regularly with the enlargement of scan rate, suggesting an excellent rate capability and good reversibility of redox procedure [46, 47]. Figure 3c shows GCD curves for the different electrodes at 1 A/g. According to Fig. 3c, the pure MWCNTs and MoS_2_ electrodes get specific capacitances of 30.4 and 90.6 F/g at 1 A/g, which are smaller than that of the MoS_2_|MWCNTs electrode. It is very interesting that the specific capacitance has increased obviously when MoS_2_ is decorated with MWCNTs. This is attributed to the good combination of conductive MWCNTs and MoS_2_ with high ionic conductivity, and the using of MWCNTs as a skeleton helps to increase the mechanical stability of the composite to prevent damage from electrical factors during charging and discharging. When the content of MWCNTs is small, the specific capacitance of MS2MWCNT1 is 132.4 F/g. With the MWCNTs percentage increasing and the mole ratio of MoS_2_ and MWCNTs reaches 1:1 in the composite materials, the specific capacitance gets 255.8 F/g at 1 A/g, which is much higher than that of the MS2MWCNT1 electrode. While the mole ratio of MoS_2_ and MWCNTs is beyond 1:1, the specific capacitance of the MS1MWCNT2 electrode begins to decrease and achieves a much smaller specific capacitance of 173.4 F/g at 1 A/g, which can be due to the small capacity of the MWCNTs. Figure 3d presents the GCD curves for the MS1MWCNT1 electrode at various current densities (0.5, 1, 2, 3, 4, and 5 A/g), which shows the specific capacitance decreases with increasing of the current density. This is because the diffusion and transfer of ions are limited at higher current density, and the lower current density can reduce the probability of destruction of the electrode material by the large electric field, so that the effective storage of charges decreases with the current density increasing. Also, the GDC curve of the MS1MWCNT1 electrode maintains a negligible voltage drop during the charging and discharging process, indicating the pseudocapacitive contribution along with the double layer contribution. The specific capacitance of MS1MWCNT1 electrode reaches 266.9 F/g at 0.5 A/g. The excellent energy storage characteristic is responsible for the large surface area and the high electrical conductivity, which provides additional electrochemical active sites and short paths for rapid ion diffusion and transport. As the current density increases to 5 A/g, the specific capacitance of MS1MWCNT1 electrode remains 203.5 F/g, demonstrating a good rate capability for the electrode. Figure 3e shows the EIS of the MoS_2_, MWCNTs, and MS1MWCNT1 electrodes to understand the impedance behavior. The enlarge EIS figure is shown as the inset. The diameter of the depressed semicircle of the Nyquist plots quantifies the charge-transfer resistance (Rct) at the electrode|electrolyte interface [48], which are found to be 4.11, 1.36, and 0.59 Ω for the MoS_2_, MWCNTs, and MS1MWCNT1 electrodes, respectively. The MS1MWCNT1 electrode shows the lowest Rct value compared to the MoS_2_ and MWCNTs electrodes, indicating that the EIS improvement for the synergistic effect of MWCNTs and MoS_2_ is helpful to decrease the energy loss at high power output. Moreover, the Warburg resistance (Wd) indicates the ionic diffusion/transport from the electrolyte to the surface of the electrode [48]. From Fig. 3e, the MS1MWCNT1 electrode exhibits a more vertical line comparing with the MoS_2_ and MWCNTs electrodes at low frequencies, which is explained that the MS1MWCNT1 electrode has a larger specific surface area, thus providing greater contact area for the electrode materials and electrolyte and helping to absorb more electrolyte [49]. Figure 3f shows the cycle stability (black line) and specific capacitance at different current densities (red line) of the MS1MWCNT1 electrode after 1000 cycles (extract a loop every 50 laps). From the black line, a small increase of capacitance is observed during the first 350 cycles, and the capacitance still maintains about 91.6% of the initial capacitance after 1000 cycles, indicating good cycling life of the composite materials [50]. The initial increase of capacitance can be attributed to gradual wetting of the electrolyte deep inside the electrode material. Then, the electrochemical active Mo sites inside the substrate electrode will be fully exposed to the electrolyte. Therefore, an increasing capacitance is displayed in the cyclic tests. Compared with other MoS_2_/carbon-based composites (Table 1), the MS1MWCNT1 electrode displays higher specific capacitance and more excellent electrochemical stability. Furthermore, the obtained maximum specific capacity is 266.9 F/g corresponding to the current density of 0.5 A/g for the MS1MWCNT1 electrode. When the discharge current density is further increased, the specific capacity decreases slowly, and 161.3 F/g is observed at 5 A/g. This is responsible for the resistance of the electrode and the insufficient Faradaic redox reaction of the active material under higher discharge current density.

**Table 1:** A comparison of the characteristics of various MoS_2_ or PANI for supercapacitors

Electrode materials	Electrolyte	Specific capacitances	Capacity retention rates	Ref.
CNTs|MoS_2_	1 M Na_2_SO_4_	74.05 F/g at 2 A/g	81% after 1000 cycles	[56]
Tubular C|MoS_2_	3 M KOH	210 F/g at 1 A/g	105% after 1000 cycles	[18]
CNTs|PANI	1 M H_2_SO_4_	113.64 F/cm^2^ at 1 mA	90.2% after 1000 bending cycles	[28]
MoS_2_|rGO|Ti mesh	PVA-1 M NaCl	17.6 mF/cm^2^ at 10 mV/s	–	[35]
MoS_2_|Mo	1 M Na_2_SO_4_	192.7 F/g at 1 mA/cm^2^	98% after 1000 cycles	[54]
MoS_2_|SiO_2_	2 M KOH	683 F/g at 1 A/g	85% after 10,000 cycles	[39]
MoS_2_ nanosheets	1 M Na_2_SO_4_	129.2 F/g at 1 A/g	85% after 500 cycles	[40]
1D PANI|2D MoS_2_	1 M Na_2_SO_4_	485 F/g at 1 mA/cm^2^	–	[51]
MS1MWCN1	0.5 M Na_2_SO_4_	255.8 F/g at 1 A/g	95.02% after 500 cycles	This work
PANI|MWCNTs	0.5 M Na_2_SO_4_	267.49 F/g at 1 A/g	94.5% after 500 cycles	This work
Quasi-solid-state MM//PM	PVA-Na_2_SO_4_	138.13 F/g at 1 A/g	–	This work

### Anode Material: PANI|MWCNTs Composite Materials

**Fig. 4:**
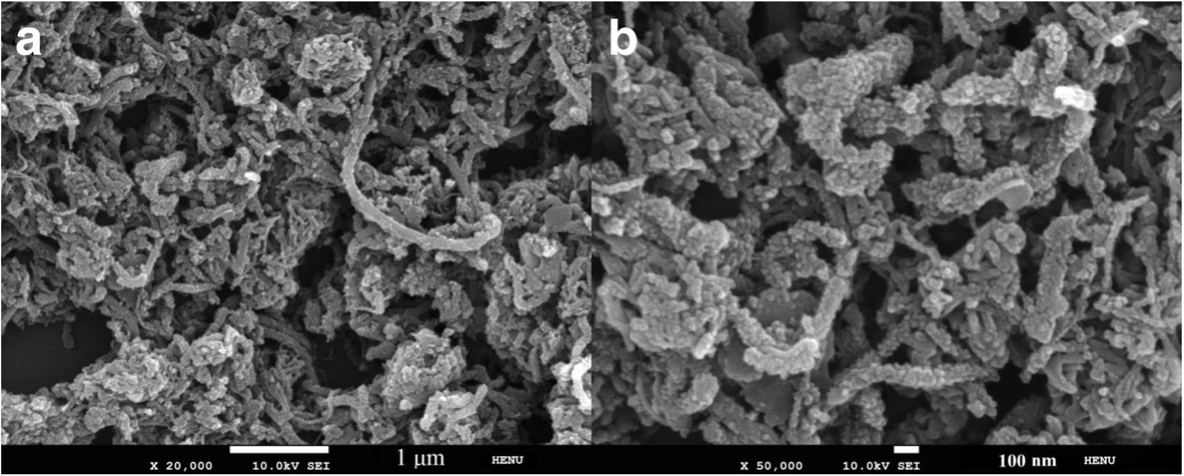
The SEM images of the PANI|MWCNTs (**a**, **b**)

Figure 4 shows the SEM images of the PANI|MWCNTs. Figure 4b is the enlarge figure from Fig. 4a. From the SEM images, it can be seen that the PANI coats on MWCNTs surface and forms a perfect organic-inorganic composite material. The MWCNTs framework helps to increase the mechanical stability of PANI and the diffusion of ions and also contributes to the improvement of the conductivity of PANI. Moreover, the pseudo-capacitance of PANI is a benefit to increase the specific capacitance of the PANI|MWCNTs composite material.

**Fig. 5:**
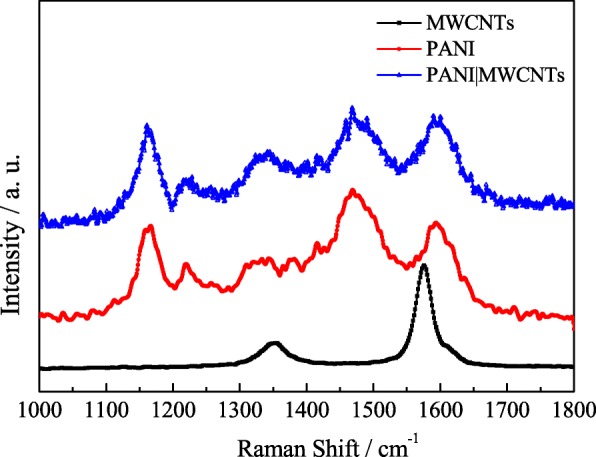
Raman spectrum of MWCNTs, PANI, and PANI|MWCNTs

Figure 5 shows several Raman peaks for the structural features of MWCNTs, PANI, and PANI|MWCNTs. Among them, Raman peaks at 1165, 1308–1347, 1468, and 1593 cm^−1^ are the characteristic peaks of PANI [51]. In the PANI|MWCNTs composite material, the characteristic peaks of PANI and MWCNTs (we have discussed in Fig. 2) are all observed though the peak signal of from MWCNT is not obvious, which is responsible for the low contents of MWCNTs and strong signals of PANI. To sum up, this result indicates that the PANI is well wrapped on the surface of the MWCNTs.

**Fig. 6:**
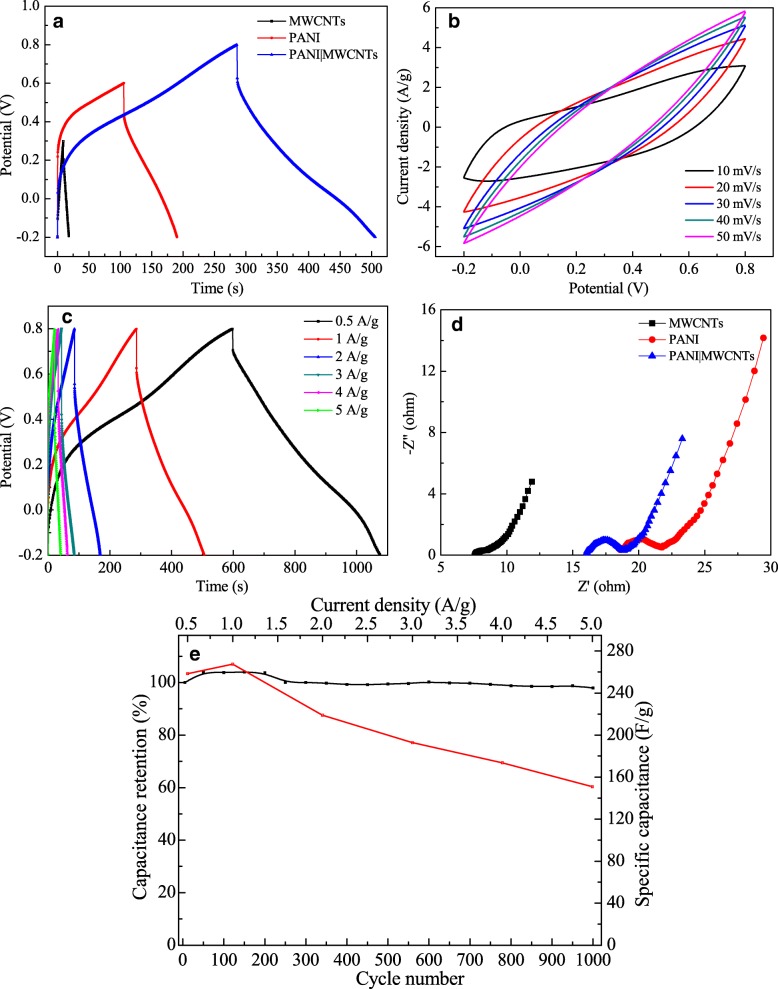
GCD curves of PANI, MWCNTs, and PANI|MWCNTs electrodes at 1 A/g (**a**); CV curves of PANI|MWCNTs electrode at different scanning speeds (**b**); GCD curves of PANI|MWCNTs electrode at different current densities (**c**); EIS of PANI, MWCNTs, and PANI|MWCNTs electrodes (**d**); the stability after 1000 cycles CVs (black line); and specific capacitance at different current densities (red line) of the PANI|MWCNTs electrode (**e**)

Figure 6a shows the GCD curves of PANI, MWCNTs, and PANI|MWCNTs electrodes at 1 A/g. From Fig. 6a, it can be seen that the voltage window of the PANI|MWCNTs electrode is much higher than that of the pure PANI or MWCNTs electrode. This may be related to the conductivity of the pure PANI and MWCNTs. This result clearly shows the improvement in voltage window for the pseudocapacitors with the MWCNTs-based composites [52]. Figure 6b shows the CV curves of PANI|MWCNTs electrode at different scanning speeds, showing the capacitive behavior of PANI|MWCNTs electrode can be greatly improved by loading of PANI. Figure 6c illustrates GCD curves of PANI|MWCNTs electrode at different current densities of 0.5, 1, 2, 3, 4, and 5 A/g, and the corresponding specific capacitance is observed to be 258.4, 267.5, 218.9, 192.8, 173.7, and 150.8 F/g, respectively, which can be calculated from Eq. (1). It shows that the discharge time is short at high current density whereas lower current density resulted in a longer discharge time. The reason why is that the slower charge-discharge rate enables the ions to have enough time for accessing the electroactive sites which are not available at higher current densities due to the time constraints of the electrolyte ions [53, 54]. In Fig. 6d, it can be seen that the Rct of the PANI|MWCNTs electrode decreases as expected compared to that of the PANI and MWCNTs electrodes. At the same time, the slope of the EIS plot for the PANI|MWCNTs electrode is steeper than that of the PANI or MWCNTs electrode in the low-frequency region. These results indicate that the PANI|MWCNTs electrode has better capacitance performance. Figure 6e shows the cycle stability (black line) and specific capacitance at different current densities (red line) of PANI|MWCNTs electrode. From the curves, it exhibits a small increase of capacitance during the first 150 cycles and remains 97.9% capacitance retention after 1000 cycles, indicating a preferable long-term cycling stability. This is because the PANI and MWCNTs nanowires can effectively increase the stability for the electrode material [55]. The red line shows the change of the specific capacity for the PANI|MWCNTs with the changing of current density. On the whole, the specific capacity decreases with the increasing current density can be attributed to the same reasons as MS1MWCNT1 electrode. Very interestingly, the specific capacity of PANI|MWCNT hybrids increases at 1.0 A/g and then decreases. This may be due to the electrode material being further activated during charging and discharging, or the electrolyte ions cannot enter certain sites of electrode materials at low current density.

### Quasi-Solid-State ASC: MM//PM

**Fig. 7:**
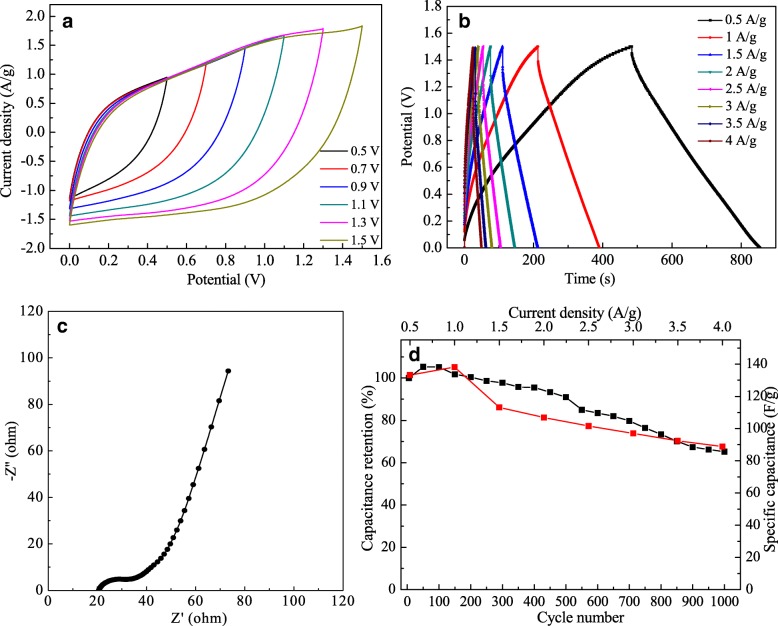
CVs at different voltage windows (**a**), GCD at different current densities (**b**), EIS (**c**), and the stability after 1000 cycles CVs (black line), and specific capacitance at different current densities (red line) (**d**) for the quasi-solid-state MM//PM ASC

Figure 7a shows that the voltage window of as-assembled quasi-solid-state MM//PM ASC can be expanded to 1.5 V. As expected, the CV curves of the as-assembled ASC can retain the quasi-rectangular shape even at 1.5 V. These quasi-rectangular curves support the quasi-solid-state MM//PM ASC with excellent capacitive behavior and reversible charging-discharging process. In order to further measure the electrochemical performance, the quasi-symmetrical shape between the discharge and charge curves ranging from 0.5 to 4 A/g were carried out and are shown in Fig. 7b, indicating that the MM//PM ASC demonstrates a good capacitive behavior with high columbic efficiency. Figure 7c shows the typical Nyquist plots for quasi-solid-state MM//PM ASC, including the solution resistance (Rs) of 20.86 Ω and charge transfer resistance (Rct) of 15.49 Ω. The low EIS of MM//PM ASC can be attributed to the introduction of MWCNTs, which results in mitigating the agglomeration of the MoS_2_ and enhancing electrical conductivity, and a conductive network connected by carbon nanotubes is beneficial to the transportation of ions and charges at the interface of electrode|electrolyte. At low frequencies, a line with a steep slope appears, indicating that MM//PM ASC has a superior capacitance behavior, which is due to the synergistic effect of double electric layer characteristics and pseudo-capacitance characteristics came from MWCNTs, PANI, and the high wettability and good catalytic of the MoS_2_. The black line in Fig. 7d shows the cycle stability of the MM//PM ASC, which also exhibits a small increase of capacitance at the initial 100 cycles and then shows a gentle downward trend and remains 65.2% capacitance retention after 1000 cycles, indicating a preferable long-term cycling stability with gel electrolyte for the MM//PM ASC. This is because the carbon nanotube skeletons and the gel electrolyte can effectively improve the stability for the MM//PM ASC. The red line in Fig. 7d exhibits the specific capacitance of the MM//PM ASC with different charging current densities. The specific capacitance decreases with the increasing of current density, indicating less active material access and reducing the effective utilization of material at a higher scan rate [20]. Among them, the maximum specific capacitance of 138.13 F/g is observed for the quasi-solid-state MM//PM ASC at 1 A/g.

**Fig. 8:**
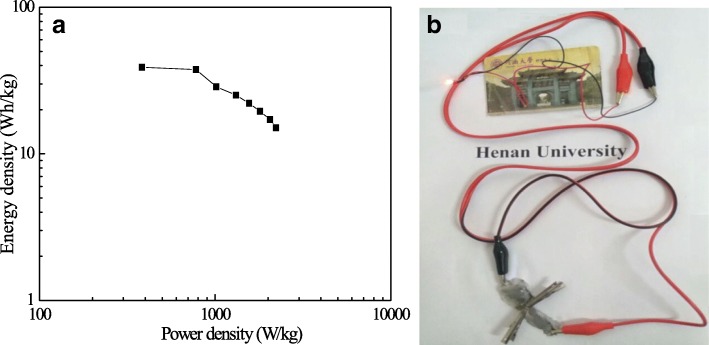
The energy density of as-prepared quasi-solid-state MM//PM ASCs at different power densities (**a**). Diagram of a small red LED bulb is lighting up with as-prepared ASCs (**b**)

The Ragone plot of the as-prepared quasi-solid-state MM//PM ASC is shown in Fig. 8a. The energy and power densities are derived from GCD at different current densities. From the Ragone plots, the quasi-solid-state MM//PM ASC shows a high energy density of 38.9 Wh/kg at a power density of 382.61 W/kg. Even at a high-power density of 2217.95 W/kg, a relatively high energy density of 15.09 Wh/kg still remains. The results illustrate that the quasi-solid-state MM//PM ASC has excellent electrochemical properties of high energy density and power output. In order to demonstrate the actual output power, Fig. 8b shows that the red LED bulb can be easily lighted based on a series group consisted of two neutral quasi-solid-state MM//PM ASCs, suggesting the potential application.

## Conclusion

In summary, two composites of MoS_2_- and PANI-mixed MWCNTs are prepared by using simple hydrothermal and low-cost chemical polymerization method and served as cathode and anode electrode’ materials for asymmetric supercapacitor devices. The specific capacitances of MS1MWCNT1 and PANI|MWCNTs electrodes are 255.8 and 267.5 F/g at 1 A/g, respectively through extensive electrochemical testing. These active composite materials not only maintain the higher stability and conductivity, but also have a larger capacity than that of a single one, which implies that the composite materials produce a better specific capacitance, higher energy, and power densities for their synergistic effect. Besides, the quasi-solid-state MM//PM ASC based on PVA-Na_2_SO_4_ gel electrolyte exhibits a good charge transfer resistance of 15.49 Ω, specific capacitance of 138.1 F/g at 1 A/g and 1.5 V, which energy density can still maintain 15.09 Wh/kg at a power density of 2217.95 W/kg. Two neutral quasi-solid-state MM//PM ASCs connected in series can light a red LED lamp. These results further indicate that this asymmetric supercapacitor has a good application prospect.

## Abbreviations

ASC: Asymmetric supercapacitor
*Cs*: Specific capacitance
CV: Cyclic voltammetry
E: Energy density
EIS: Electrochemical impendence spectroscopy
GCD: Galvanostatic current charge-discharge
MoS_2_: Molybdenum disulfide
MWCNTs: Multi-walled carbon nanotubes
PANI: Polyaniline
Rct: Charge transfer resistance
Rct: The charge-transfer resistance
Rs: Solution resistance
SEM: Scanning electron microscopy
*η*: Power density
